# Supplementary material for: Benefit of sequential bilateral cochlear implantation in children between 5 to 18 years old: A prospective cohort study
Source: PLoS One. 2022 Jul 28;17(7):e0271497. doi: 10.1371/journal.pone.0271497 (PMC9333257; doi:10.1371/journal.pone.0271497)
Supplement: S1 Fig — β unilateral = -0.23 (n = 29); p = 0.22. β bimodal = 0.08 (n = 28); p = 0.66. Note: CI1 = first cochlear implant; CI2 = second cochlear implant CVC = consonant-vowel-consonant (speech perception). (DOCX) [file pone.0271497.s001.docx]

**S1 Fig. Regression line between speech perception scores (CVC) in quiet in the bilateral CI situation and the inter-implant interval.**


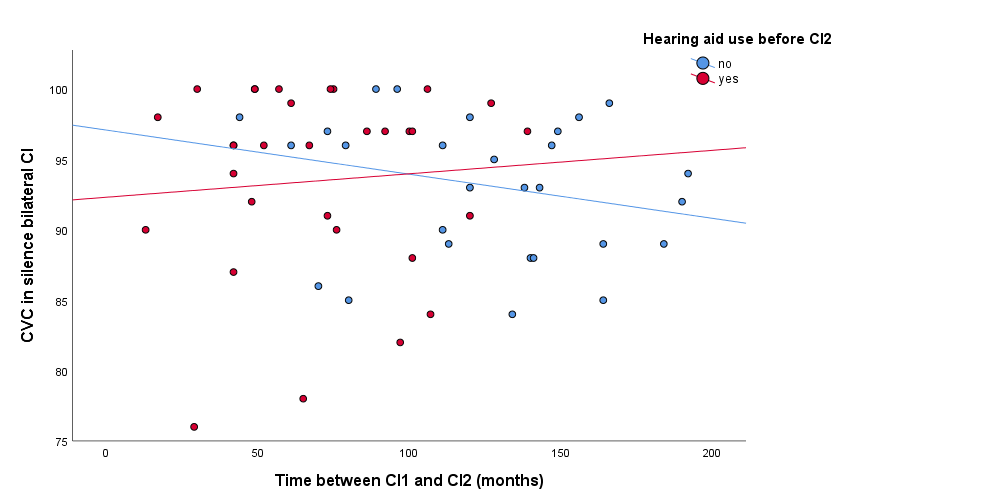


β unilateral = -0.23 (*n* = 29); *p* = 0.22. β bimodal = 0.08 (*n* = 28); *p* = 0.66. *Note:* CI1 = first cochlear implant; CI2 = second cochlear implant CVC = consonant-vowel-consonant (speech perception);
