# Supplementary material for: Benefit of sequential bilateral cochlear implantation in children between 5 to 18 years old: A prospective cohort study
Source: PLoS One. 2022 Jul 28;17(7):e0271497. doi: 10.1371/journal.pone.0271497 (PMC9333257; doi:10.1371/journal.pone.0271497)
Supplement: S2 Fig — β unilateral = 0.44 (n = 17); p = 0.63. β bimodal = 1.49 (n = 21); p = 0.34. Note: CI1 = first cochlear implant; CI2 = second cochlear implant CVC = consonant-vowel-consonant (speech perception). (DOCX) [file pone.0271497.s002.docx]

**S2 Fig. Regression line between speech perception scores (CVC) in noise in the bilateral situation and the inter-implant interval.**


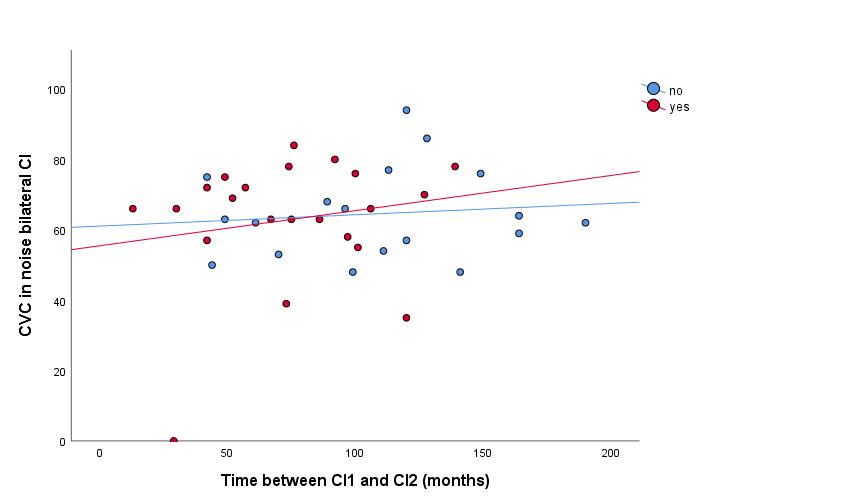


β unilateral = 0.44 (*n* = 17); *p* = 0.63. β bimodal = 1.49 (*n* = 21); *p* = 0.34. *Note:* CI1 = first cochlear implant; CI2 = second cochlear implant CVC = consonant-vowel-consonant (speech perception).
