# Supplementary material for: Benefit of sequential bilateral cochlear implantation in children between 5 to 18 years old: A prospective cohort study
Source: PLoS One. 2022 Jul 28;17(7):e0271497. doi: 10.1371/journal.pone.0271497 (PMC9333257; doi:10.1371/journal.pone.0271497)
Supplement: Attachment — Submitted filename: Rebuttal letter PONE-D-19-15922.docx [file pone.0271497.s003.docx]

| **Reviewer 1** | **Response** |
| --- | --- |
| Over the whole paper you speak from first Implantation (CI1) early in life. In table 1 is written that age of CI1 implantation (median in years) is 3 (0-13). From my point of view is it not an early in life implantation. Early in life would be for all children under the age of 2. So it is necessary to change all the text passages in which you wrote „early in life“. | We thank the reviewer for the opportunity to further elaborate on this subject. The cohort of children included in this manuscript were all having prelingual deafness including children with progressive hearing losses. They were implanted with their first implant at a relatively early age (median 3 years). We agree with the reviewer that the quote ”early in life“ could be misleading and changed this to ‘relatively early age’ in combination with the statement of the median age. |
| List of abbreviations: HA is missing, it is used in table 2. | We have included ’hearing aid’ (HA) in the list of abbreviations. |
| I don’t understand table 2. Which are the data preoperatively and which of them are measured postoperatively e.g. CVC score in quiet (daily life) - what does it mean? Are this data preoperatively? If yes, I don‘t understand why you have a score for CI2 in this line? The Food Note 1- Daily in life- pointed out of hearing aid use. | During the revision we have changed the content and lay-out (including column titles) of Table 2 to improve the comprehensibility. Also, we specified the subtitles in the table and added additional information in the Foot Note to clarify the testing conditions used for the data presented in this table. |
| You did not find an association with the speech perception scores and the inter implant interval or age at CI2. It is possible that your big age range for CI1 influences this result. You have overlapping age ranges for age at first and second CI. If a child got their first CI with 10 years and the second CI 5 years later, you will not find differences in the scores between both ears although the child has a long inter implant interval.  Additionally, Illg et al. (2017) found a group of children which had no differences between speech compre-hension results of first and second CI and defined the optimal interval. It would be helpful to discuss this further. | We thank the reviewer for the opportunity to explain our statement. Based on these remarks we added data of preoperative residual hearing levels of the second CI ear to the manuscript. By this, we were able to analyse more in detail the influence of residual hearing as well as the inter-implant interval and age of implantation of the second CI ear on performance outcomes. This analysis resulted in the demonstration of a significant and relevant improvement in speech perception in both quiet and noise scores at 12 months post-implantation compared with the (best aided) unilateral CI situation Though, no statistical significant effect was seen of residual hearing, inter-implant interval and age of implantation of CI2 on speech perception outcomes (Table 4). To explain this outcome, we revised the discussion and included the study of Illg et al. (Discussion lines 407-417) |
| SSQ: line 403-409: Please discuss this briefly with additional literature and mention if this questionnaire is used by other authors in sequential bilateral children/ adolescents. | Information about subjective perceived speech perception, localization abilities and quality of sound was collected with the parent-reported Speech, Spatial and Qualities of Hearing Scale (SSQ). This questionnaire consists of 30 questions distributed in 3 domains: speech, spatial and qualities of hearing. The questionnaire is adjusted for children by Galvin et al. and was translated in Dutch. We added this information to the methods section. See line 222-230. |

| **Reviewer 2** | **Response** |
| --- | --- |
| This study is of interest and well-motivated. As the authors point out, there are benefits and limitations because of the multicenter approach, the greatest limitation being missing data. Although the total study population is reported as n=85, from Fig 1 it is clear that, at most, there were only 63 who had both pre- and post-CI2 data on any given measure and at most only 9 for the SSQ. 10 children became non-users of CI2. That leaves 12 who received the CVC in silence pre-CI2 but not post-CI2. Presumably results for the other measures are a subset of the 63. It is misleading to include so many children who were not tested at both intervals. It is helpful to include the information about those who became non-users; however, it would be more helpful if the Table 1 only included information for participants with data at both intervals and baseline characteristics of the non-users reported in a separate table or column. | We thank the reviewer for this positive statement and encouragement to make the data more transparent. We agree with the reviewer that the numbers of patients per outcome measures needed clarification.  Therefore, we changed the passages in the results about the total study population and included Figure 1 at the start of the result section with numbers categorized by outcomes. (See results section, line 253-259). Secondly, we added the number of data used for each specific outcome measure during the text, see line 280-295. In addition, we added in Table 1, Table 2 and Table 4 the n=x to clarify the amount of data which is used in the analysis. Furthermore, we added the biographic data of the non-user group in Table 1 and highlighted the issue of missing data in the discussion section (line 436-438). |
| The authors indicate randomly missing data were handled by multiple imputation. It would be more appropriate for the Wilcoxon signed rank test to only include results for which data was collected. | Data did not comply with the assumptions for missing data at random, therefor multiple imputation was not possible. To clarify this for the reader we added this to the results section (results, line 255-256). As suggested, the Wilcoxon signed rank test was applied for comparisons of related samples as discussed in the methods section (line 244-245) and indicated in the legend of the outcomes table 2 for clarification. |
| The range of age for CI1 extends to age 13 years which is not considered a young age for cochlear implantation and including participants that old likely confounds the results. I suggest using a younger age when selecting participants to include in the analyses. | We thank the reviewer for this suggestion. The cohort of children with bilateral hearing loss who were implanted unilaterally earlier in life comprised of children with prelingual deafness and children with progressive hearing loss. Indeed, they received their first implant between 0 and 13 years of age. To highlight this issue, we revised the phrasing of ‘early life’ as stated in the comments of reviewer 1. All in all, we believe that our data represents the clinical setting with children with progressive hearing losses and not all implanted with their first CI within the very first years of life. This makes the results applicable for use in a clinical setting and useful for counselling. We therefore revised the discussion in the strengths and limitations section (line 428-435 and 438-442). |
| Although for the most part the English used was clear, the manuscript would benefit from editing by a native English speaker who is familiar with the area of study. There are several places where the intended meaning is unclear. | We have asked a native speaker for a revision to correct readable English. |
| For the pediatric and adult CVC measures, were participants given the same measure at both test intervals? If so, please clarify. Was the word or phoneme score used? | The measurement of speech recognition was performed with two randomly selected CVC word lists, taken from the available pediatric (patients < 12 years of age) or adult (patients ≥ 12 years of age) word list of the NVA (Dutch Audiology Society) according to age at the time of evaluation. For each tested condition, the mean percentage correct phoneme score was calculated over the two lists of words. The paragraph detailing this test was adjusted for clarification (lines 190-200) |
| The manuscript would also benefit from greater input from a speech language pathologist to address the following: The rational for including the recalling sentences subtest of the CELF – was it the only subtest given? The authors indicate that the CELF was given to test expressive speech development – the CELF is a language measure and not a speech measure. The CELF is designed to test language proficiency in young children not subjects (line 207) – in other words, it is not a research specific tool but rather a clinical tool. Throughout the authors should revise “development of speech and language” to include only language because speech skills were not evaluated. | We added new information in lines 212-219 to explain the usage of the subset “recalling sentences” of the Dutch version of the Clinical Evaluation of Language Fundamentals (CELF-4-NL). The CELF-4-NL is designed to represent the language proficiency in young children of 5 to 18 years old. In this subtest the patient had to listen to spoken phrases uttered by a speech and language therapist, concerning sentences of sequential increasing length and complexity. Thereafter, the patient had to repeat the phrases in the same order and in the correct sentence structure. Outcomes were reported in norm scores, calculated with a reference score, according to age. This subtest of the CELF-4-NL requires higher order complex linguistic skills. It is a validated test and outcomes are corrected for learning effects.  We also discussed this subject and outcomes in more detail in our revised discussion (line 385-401). |
| In Table 4 the n for gender is 85, but the outcome measures were not obtained on nearly that many children. This is misleading and should be corrected. | We apologize for this error. In addition, we now added the number of available data per variable and outcome measure in Table 4. |
| The introduction doesn’t seem to include articles published after 2015 or 2016. A search of “Bilateral cochlear implant” and “sequential” published in 2016 or later brings up more than 30 articles. It seems that at least some of those might be pertinent to this manuscript. | We revised the included references in the introduction and the discussion to cover the most recent insights on this matter. |
| The factors described as those indicating limited expectations and an exclusion factor were confusing. Severe mental retardation should be a factor of comorbidity. Psychomotor retardation should be defined (in a google search it comes up as something most common in adults). There were psychologists on the teams – did they perform IQ or developmental testing? Generally, the term developmental delay is currently used more commonly than retardation and may be less offensive to some readers. | We thank the reviewer for the suggestions. Comorbidity is now categorized in somatic comorbidity, a subgroup of psychiatric and behavioral problems and a subgroup of developmental delay including mild cognitive impairment (see methods section, line 167-169). |
| Speech Language Pathologists rather than therapists is preferred. | We thank the reviewer for this suggestion and we changed it in our manuscript (see line 142 and 190). |
| I am unfamiliar with using “e.c.i.” with congenital hearing loss – please define or spell out. | We have defined the abbreviation as ‘with unknown cause’ (e causa ignota). |

| **Reviewer 3** | **Response** |
| --- | --- |
| The manuscript reports significant improvements in speech recognition in quiet and in noise following receipt of the second implant. This is not a significant advancement for the discipline as a body of evidence already exists indicating that providing bilateral stimulation compared to unilateral stimulation is beneficial to children’s speech perception. Furthermore, this work has been conducted in larger samples with prolonged longitudinal follow-ups in comparison to the current study.  The manuscript also claims that there was no significant benefit of sequential implantation on language abilities or on subjective hearing abilities. However, the outcome measures chosen, availability of data, and statistical power and analyses employed are too inadequate to make or support these main claims. | We thank the reviewer for the opportunity to further elaborate on this subject. Generally, speech recognition scores in quiet were considered to be good in the bilateral CI condition in accordance with previous literature. Though in this study, a significant increase of speech recognition in noise after the second CI was demonstrated even with a mean inter-implant interval of 8 years and a median age of 12 years [SD = 4,0 years] at time of the second implantation. This outcome adds value to existing literature describing benefits in situations with shorter intervals and younger study populations.  The strength of this study is the unique study population of sequentially implanted children with the first implant at a relatively early age (median 3 years) and their second implant between 5-18 years of age, also encompassing children with progressive hearing losses. By the multicenter design and prospective data collection the outcome of this study will strengthen the evidence of the benefit of sequential cochlear implantation with larger implant-intervals in children. In addition, in this study we were able to combine objective and subjective outcomes of hearing abilities. This makes the results applicable for use in a clinical setting and useful for counseling patients and their parents about the benefit of this intervention, even with children at an older age. We highlighted the implications of the selected study population and resulting outcomes more in detail in the discussion section. |
| The literature review/introduction is insufficient and key work is not considered. This appears to be a study of a small cohort of children in the Netherlands who were granted a “conditional reimbursement”. It is therefore unclear how the current study fits in the context of previous literature and what contribution it makes in terms of knowledge or impact on clinical practice. Furthermore, no context is given to claims of an association between use of a contralateral hearing aid and language outcomes. | We thank the reviewer for the critical statement and we made a major revision of the introduction part (line 101-109) and explained the context more thorough in the discussion part (line 385-401).  Children from our study cohort with demonstrated effectiveness of the first implant were thought to qualify for a second (contralateral) cochlear implant. This was substantiated by earlier evidence showing the advantage of sequential bilateral implantation for speech recognition in quiet and noise and the receptive vocabulary. In the long term, the improvement in the more basic language skills, like receptive vocabulary, may also lead to an improvement in more complex linguistic skills. |
| I have several concerns over the outcome measures chosen and the suitability of the analysis methods employed. Assessment of speech recognition in quiet and noise is standard in the field, however, only their ability to perceive monosyllabic words is assessed. What about sentence recognition? Furthermore, use of the CELF-4 ‘recalling sentences’ subscale is wholly insufficient as a measure of “language skills”. This is essentially a speech perception and recall test, and does not tap into language abilities such as understanding of linguistic concepts, morphological rules, semantics, comprehension etc. The authors also provide no justification for why only this one sub-scale was chosen to represent their language ability. | We thank the reviewer for the room to explain our statement, which we now discuss in more detail in the method (see line 190-221).  The subtest ‘recalling sentences’ of the CELF-4-NL was used. This test requires higher order complex linguistic skills to be used. The CELF-4-NL is validated with other vocabulary tests, including the PPVT-III-NL and secondly corrected for learning effect. Those two tests are furthermore used as a standard use of care in the clinical setting in The Netherlands. |
| Overall, analysis techniques employed are inappropriate and inadequately described. For instance, linear regression analysis is performed on 15 independent variables and three dependent variables. This analysis should only be used when assessing the relationship between one independent and one dependent variable. Multivariate linear regression could be used to assess the impact of these 15 variables on ONE of the outcome variables, however the sample size is too small for this number of variables being investigated. | We thank the reviewer for the critical statement. Univariable linear regression analyses was performed to determine the effect of individual variables on performance outcomes. Multivariable regression analysis was not performed as no significant associations were found by univariable regression analysis and the number of data was too small for a correct multivariable analysis. In addition data did not comply with the assumptions for missing data at random, therefor multiple imputation was not possible. To clarify this to the reader we revised the ‘data analysis’ section (245-249) and parts of the ‘linear regression analysis’ paragraph (lines 355-362). |
| Although 85 children were implanted, post-CI2 data is only available on a subset of these enabling pre- post-CI2 comparisons in a small sample (speech and language n=24, quality of life n = 9), with insufficient power to conduct the statistical tests required in revised form. I also do not believe the study to have sufficient potential due to inadequate operalization of speech and language outcomes through a poor and unsubstantiated choice of measures. | We want to refer to our previous statements above. Outcome of the described cohort is of potential interest by the great ranges of implant intervals described and a relatively high age at time of the second cochlear implantation. The study cohort also contains children with progressive hearing loss which makes it an unique study population. The outcomes used in our study are standardised clinical test. Indeed, as suggested by the reviewer, the outcome of the study is limited by missing data. We highlighted the strengths and limitations more in detail now in the discussion section. Although a part of the population became non-user, this is of further interest to the reader to hypothesize about the reasons for this. We revised this section into discuss this in more detail (lines 415-to 424). |
| The authors do not provide access to their data. They state that this is due to privacy reasons (medical data). I do not understand this the anonymized subset of data presented in the manuscript should be able to be publicly shared. I.e. speech perception and quality of life scores, age at implantation, inter-implant delay. These are standard data that are typically made available either in Tables within the publication or in an online repository. I would suggest further justification and details for why the relevant and anonymized data cannot be shared. | At this time all data are fully available without restriction. Data can be provided on request by contacting author Adriana L Smit, MD, PhD, Department of Otorhinolaryngology, Head and Neck Surgery, University Medical Center Utrecht, PO Box 85500, 3508 GA Utrecht, The Netherlands; E-mail: A.L.Smit-9@umcutrecht.nl |
| It is written in a logical order and written clearly (although not comprehensively enough). There are several grammatical errors throughout the manuscript. If this is due to it being written in a non-native language, the Authors should have the manuscript proof-red by a native-English speaker first. | We thank the reviewer for this positive statement and encouragement. We have asked a native speaker for a revision of the sentences in correct readable English. |
