# Supplementary material for: Benefit of sequential bilateral cochlear implantation in children between 5 to 18 years old: A prospective cohort study
Source: PLoS One. 2022 Jul 28;17(7):e0271497. doi: 10.1371/journal.pone.0271497 (PMC9333257; doi:10.1371/journal.pone.0271497)
Supplement: Attachment — Submitted filename: Response to Reviewers.docx [file pone.0271497.s004.docx]

Dear editor,

We would like to thank the editor for the opportunity to resubmit the manuscript entitled ‘Benefit of sequential bilateral cochlear implantation in children between 5 to 18 years old: a prospective cohort

study’.

We submitted this paper last year and received valuable comments of the reviewers of PLOS ONE with the advice to revise the paper thoroughly before publication could be considered. Based on these comments we revised the paper carefully to improve the quality of the text including the statistical analysis and conscientiously revised the discussion. This resulted in an improved manuscript which we would like to offer to PLOS ONE for reconsideration of publication. Our detailed responses to the reviewers’ comments of the previous submission can be found below.

Thank you for considering our manuscript for publication.

Sincerely,

On behalf of the author-team,

Drs. W.J. Kleijbergen

| **Reviewer 1** | **Response** |
| --- | --- |
| The authors state that “so far, studies of sequentially implanted children included populations with a mean age between 5 to 10 years” and that outcomes “in older children and adolescents and the influence of preoperative residual hearing is not clear from the literature so far”. This is not the case and the authors are clearly unaware of the available body of literature. Of particular relevance, please see key studies from Dr Karen Gordon’s lab such as Easwar et al., (2018) and Polonenko et al., (2018). These papers include data from older children and adolescents and demonstrate the bilateral benefits of sequential implantation in quiet and noise. Furthermore, they demonstrate the relationship between inter-implant delay and pre-operative residual hearing (amongst other key factors such as age and daily CI use). | We thank the reviewer for the feedback. We agree that there is a body of evidence on factors in relation to hearing outcome in sequentially implanted children such as interplant delay and pre-operative residual hearing. To provide information to the reader with evidence on this matter we revised the introduction and added these references.  Even in the high-quality studies of Polenko et al, only a few cases were bimodally fitted prior to receiving a second (sequential) cochlear implant (n=18 implanted sequentially with bimodal stimulation versus n=170 implanted sequentially with unilateral stimulation during inter-implant delay).^[1]^ Still, current literature on outcomes and the influence of patient related characteristics in older children and adolescents is scarce. We revised the introduction to highlight this issue and clarify this to the reader. |
| It is not until the methods section that is becomes apparent that some children in the sample used a unilateral CI only and so experienced unilateral deprivation/stimulation prior to receiving the second CI. Whereas others (45% stated in Table 1) wore a contralateral hearing aid and so experienced bimodal input (i.e. bilateral access to sound from a combination of electric and acoustic input). These are two very different groups and this should be highlighted to the reader from the very beginning of the manuscript. Literature on bimodal users should also be reviewed. The definition of inter-implant delay therefore also differs for these groups and should be explicated for the non-expert. I.e. duration of unilateral deprivation/stimulation, vs duration of bimodal stimulation. Given that almost half of the sample were bimodal CI and HA users and the other half were unilateral CI users, this should be treated as a factor in the analyses. Bimodal sequential users are likely to receive more benefit from a second implant as their pathways have received acoustic stimulation vs no stimulation in the unilateral CI users. There is evidence that bimodal sequential users gain similar bilateral advantage to that of simultaneous bilateral CI users. Therefore, data for unilateral sequential and bimodal sequential should be investigated to understand if there are group differences here as this could be driving the main result reported here. | We agree with the reviewer that this issue it is not clearly enough explained in the manuscript. The cohort of children with bilateral hearing loss who were implanted unilaterally early in life indeed comprised of children with unilateral and bimodal experienced input.  We now added this to our introduction (line 111-113), method (line 142-143), results section (line 303-306) and our discussion (line 435-439 and 493-496). We also added information of these subgroups to Table 2 to clarify the differences between the implanted children.  We performed an univariable regression analysis to evaluate the impact of unilateral vs. bimodal stimulation on outcomes (Table 4). The outcome showed no significant relation with overall measures of performance and therefore was not further elaborated, as described in line 379-389. While our cohort is substantial, it was not possible to perform a multivariate analysis; the missing data on some variables made our possibilities for an in depth statistical analysis limited. For example, ‘hearing aid use’ was only recorded in 42 patients. Performing multivariable analyses will therefore introduce overfitting, and therefore result in unreliable outcomes.  We would like to refer to the article of Groenwold et al.^[2]^ that elaborate more on the topic of small sample sizes and bias. |
| Age at time of speech perception testing should be entered as a covariate as younger children can have worse speech reception thresholds, and any developmental effects should be considered when reporting changes in children’s speech and language outcomes over a 12-month period. | The Dutch phoneme CVC test consists of two versions: for young children (up to 6-7 years of age) and one for children starting age 7 and adults. The children testing lists stem from the same material but are a selected sub-section and less phonetical balanced. There is no age correction for the developmental effects mentioned by the reviewer.^[3]^ In clinical practice it is seen that young children can reach high scores, so a possible age-effect is considered to be limited or very small. |
| I am concerned that the objective of the manuscript is to determine the benefit of a second sequential CI, yet “limited expectations in terms of speech perception results were an exclusion factor to qualify for this study”. Therefore, the inclusion criteria could bias the results by including only those children who were expected to show improvements in speech perception. It is understandable to exclude children based on non-verbal communication/inability to undergo speech perception outcome measures. However, if they received little benefit from the first implant, is that to say that they would not benefit from bilateral input? | We agree with the reviewer that a second cochlear implant could be considered also in children communicating (mainly) non-verbally or even in children with limited outcomes of the first cochlear implant. Therefore, counselling and information about expected outcomes is of importance before progressing to a second implantation. Though, in this study, the used eligibility criteria were based on the criteria advocated by the Dutch health care institute for conditional reimbursement at that time. These criteria have impact on the generalizability of the outcome of the study. To clarify this for the reader we rephrased the methods section (line 151-156) and added this information to the limitation section (line 471-475). |
| Non-use of the second CI is an important outcome as it suggests that the child gained no benefit from sequential implantation. The outcome data from these children is not missing at random – it is missing because they have low benefit. The authors acknowledge this and provide information about this (lines 330-351). However, rather than repeating summary statistics already listed in Table 1, it would be useful if the authors provided information regarding any significant differences between the non-users and the user groups. Of note, the non-users appear older at CI2, have a longer inter-implant interval, and only 10% compared to 45% of users are bimodal hearing aid users prior to CI2. The implications of this on the current findings and main claims should be considered and unpacked further. | We decided to present the data about the non-users descriptively because the group of non-users was too small to perform adequate statistical analysis to compare the non-users to the user group. We agree with the reviewer that understanding the reasons underlying this non-use are of upmost importance. Therefore, we mention this important issue in the section *Future recommendations* (line 487-496). |
| The authors state that there are no significant associations between outcomes and certain predictors of interest. However, they fail to identify and discuss some clear trends that are approaching significance. It is likely that some associations have not been detected due to my aforementioned concerns of the mixed group of bimodal sequential and unilateral sequential users, as well as age at time of testing that should be treated as a covariate. Indeed, Table 4 shows that hearing aid use is a predictive factor of language outcomes. The relationships presented in Table 4 should be examined in these two groups separately. Data should be presented visually in the form of scatterplots with regression lines, for example, so that any trends and their importance can be assessed by the reader. | Ideally, our cohort was applicable for a strong multivariate analysis in which also the bimodal users and unilateral cochlear users could be analysed separately. Actually, the amount of data in our study was too small to perform correct, statistically significant analysis on this subject. But, the described benefit of sequential cochlear implantation with larger implant-intervals in children, as described in our multicentre study with prospective data collection, will strengthen the evidence on this matter. In addition, in this study we were able to combine objective and subjective outcomes of hearing abilities. This makes the results applicable for use in a clinical setting and useful for counselling. To present the data visually we added a scatterplot about speech perception outcomes with regression lines in the Supporting information and added this information to line 384-388. |
| *Specific comments:* |  |
| Line 65. “contralateral” implant – it is not clear what it is contralateral to (although I assume the first implant). Using “second implant” would be clearer for a naïve reader.  72. ...the secondary outcomes WERE disease...  74 ...were compared USING THE WILCOXEN SIGNED-RANK TEST.... | We changed the text accordingly. |
| 74 statistical association between what? Outcomes and clinical characteristics of the CI user? Please specify. | We specified this text accordingly. |
| 84. 1-16 years of inter-implant delay is a very large range that includes children with minimal and short duration of delays as well as long delays. However, the authors classify it here as a “great inter-implant interval” with the main claim being that benefit can be obtained despite a great interval. However, it is possible that these benefits observed are being driven by those with minimal and short delays. This is difficult for the reader to assess given the absence of plots for data visualization. | Because of the wide range in the inter-implant interval we used the median instead of the mean values. By the univariate analysis no statistically significant effect was seen for the studied cohort by the length of the inter-implant interval on the measures of performance for the studied cohort. In addition, it is possible that bimodal sequential users perform better in de bilateral cochlear situation compared to the unilateral sequential users which therefore made the effect of inter-implant delay not significant clear. To take this knowledge into account we added this information to the results section with a scatterplot in the Supporting information (line 384-388). |
| 97 – conditional reimbursement – please briefly explain what this means in this context | We elaborated the text on this issue (line 100-102) |
| 100 – what does “minor” hearing performance mean? Poorer speech perception/worse outcomes compared to short-delay or simultaneously implanted? | We changed this part of the paragraph (line 106-125) and therefore this text was erased from the manuscript. |
| 157 – if describing male or female then please refer to “sex” rather than “gender”, unless they were asked what they gender identify with  Table 4: “Gender, Men, Woman” should read “Sex, Male, Female” | We changed the text accordingly throughout the manuscript. |
| 157 – age at time of speech test should also be reported for pre and post measures | We changed the text accordingly. |
| 176 – please outline how frequency of CI use was determined. Was this self-report or from datalogging built into the CI device? | This was self-reported by the CI users/parents. We added this information to line 197/198. |
| 184 – 186. Some children wore a unilateral CI only and so experienced unilateral deprivation/stimulation prior to receiving the second CI. Whereas others wore a unilateral CI with a contralateral hearing aid and so experienced bimodal input (i.e. bilateral access to sound from a combination of electric and acoustic input). These are two very different groups and this should be highlighted to the reader from the very beginning of the manuscript. Furthermore, literature on bimodal users should also be reviewed. The definition of inter-implant delay therefore differs for these groups and should be explicated. I.e. duration of unilateral deprivation/stimulation, vs duration of bimodal stimulation. | We rephrased the text about unilateral versus bimodal stimulation as explained above. |

| **Reviewer 2** | **Response** |
| --- | --- |
| So far, I am still missing a clear research question as far as the main message is concerned. The paper contains a collection of speech comprehension data for the patient group. These have also been presented in dependence of predictors in other papers (e.g. Illg et al. 2019, which was also cited). | To clarify the study aim we added this information to the material and methods section. (line 133-136) |
| Page 4, line 110 et seq. is described: "The speech perception outcome of sequential BICI in older children and adolescents and the influence of preoperative residual hearing is not clear from the literature so far" What do you mean with this sentence? What is the point? | We changed the text accordingly (line 122-128). |
| In the evaluation, the 10 non-users are listed separately to describe the group. However, their speech perception results are not included in the calculations. When conducting a prospective study, these data would also have to be calculated, or did these patients no longer appear at the 12-month deadline? It is not clear which result in speech understanding is achieved by a non-user, because there are different reasons for this. However, these data also belong to the total group and must not be eliminated, according to my understanding. | In the 12-month evaluation it was recorded if the patient was user or non-user of the second CI. In case of non-use, their speech perception results were not reported in the database because these children did not wear the second implant, so were not used to have hearing experience with the new implant.  In case of missing data multiple imputation is a statistical method that can be used to make estimates more precise. However, the data was not at random missing and did not comply with the assumptions for missing data at random. Therefore, multiple imputation was not possible for these missing data. We adjusted the text to make this more clear (line 252-254). Additionally, the Wilcoxon signed rank test was only performed on the available collected data. |
| I also find it very difficult that patients with hypoplasia of the cochlea and the like were included in the total group, because they have different preconditions than children and adolescents with regular anatomy. From my point of view, these two groups should be separated and considered separately. | We agree with the reviewer that children with inner ear malformations could be seen as a distinct group by underlying syndromes, or the necessity for deviations from standard surgical procedures / selected electrodes for implantation. Though, patients ‘with cochleovestibular malformations that might prevent full insertion of electrode arrays’ as well as patients with comorbidities (such as developmental delay) limiting functional outcomes were not eligible for this study. In addition, the children included in our cohort with inner ear malformations, such as cases with Enlarged Vestibular Aqueduct [EVA], were eligible for our study because they demonstrated effectiveness of the first implant on hearing performance and were therefore included for receiving a second (contralateral) cochlear implant device (see line 308-310). The results on performance of these children is useful in the future to support the clinical counselling. |
| Furthermore, I do not understand in table 2 the CVC score in quiet (no HA) values for the bilateral CI condition and likewise the CVC scores in noise (no HA) in bilateral condition. Why do the have the same values like in daily life? If these are the results of a measurement without HA, then unilateral measurements must have been taken, or? Why is this listed under bilateral condition? | We agree with the reviewer that the presentation is unclear. We adjusted to table to clarify the comparisons made. (Table 2) |
| The description of tabel 1 (page 12 line 279 et seq.) is partly redundant because of adding the table. | We agree with the reviewer and revised the paragraph to highlight the most important results and present additional information to the numbers provided in Table 1 in this section (line 295-298). |
| Please correct the following sentences page 18, line 400 "language skills might be eventually seen in de the ....." | We added new information in line 426-428. |
| My request for the further revision is that a clear question emerges on which the discussion can also be guided and that the data evaluation takes place in clearly defined groups. | To clarify the research question and the implications of the study and study outcomes we revised the text by adding explicitly the objective to the methods section (line 133-136), the handling of data of cases with inner ear malformations and bimodal stimulation vs. unilateral CI before the second implantation (line 144-156) and highlighted the implications of these outcomes (discussion section) to the reader. |

**Supporting information**

**S1. Fig 2a Regression line between CVC in silence in the bilateral situation and the inter-implant interval**.

*Note:* CI = cochlear implant; CI2 = second cochlear implant CVC = consonant-vowel-consonant (speech perception)

**S2. Fig 2b Regression line between CVC in noise in the bilateral situation and the inter-implant interval.**

*Note:* CI = cochlear implant; CI2 = second cochlear implant CVC = consonant-vowel-consonant (speech perception)

1. Polonenko MJ, Papsin BC & Gordon KA. Limiting asymmetric hearing improves benefits of bilateral hearing in children using cochlear implants. Sci Rep 2018;8:13201. https://doi.org/10.1038/s41598-018-31546-8
2. Groenwold RHH, Klungel OH, Altman DG, et al. Adjustment for continuous confounders: an example of how to prevent residual confounding. CMAJ Can Med Assoc J. 2013;185:401–406. doi: 10.1503/cmaj.120592
3. Bosman AJ, Smoorenburg GF. Intelligibility of Dutch CVC syllables and sentences for listeners with normal hearing and with three types of hearing impairment. Audiology. 1995;34(5):260–84.
