# Supplementary material for: Benefit of sequential bilateral cochlear implantation in children between 5 to 18 years old: A prospective cohort study
Source: PLoS One. 2022 Jul 28;17(7):e0271497. doi: 10.1371/journal.pone.0271497 (PMC9333257; doi:10.1371/journal.pone.0271497)
Supplement: Attachment — Submitted filename: Response to Reviewers.docx [file pone.0271497.s005.docx]

Dear editor,

We would like to thank the editor for the opportunity to resubmit the manuscript entitled ‘Benefit of sequential bilateral cochlear implantation in children between 5 to 18 years old: a prospective cohort

study’.

We re-submitted this paper last year and received valuable comments of the reviewers of PLOS ONE with the advice to revise the paper again before publication could be considered. Based on these comments we revised the paper carefully to improve the quality of the text including the statistical analysis and reconstructed Table 2 and 4 in detail. This resulted in an improved manuscript which we would like to offer to PLOS ONE for reconsideration of publication. Our detailed responses to the reviewers’ comments of the previous submission can be found below.

Thank you for considering our manuscript for publication.

Sincerely,

On behalf of the author-team,

Drs. W.J. Kleijbergen

| **Reviewer 2** | **Response** |
| --- | --- |
| Please revise the references in the text. In the chapter Discussion, for example, the references do not have associated numbers, so that one can then find them. | We thank the reviewer for the feedback. We revised the references and citationnumbers, and now these are up to date with associated numbers. |
| P. 13 line 357 The dot from the end of the sentence belongs after the bracket (Table 1). | We replaced the dot in the sentence. |
| The last two sentences in the Conlcusion section are slightly misleading, please adjust them. First you write that the inter-implant distance has no significant influence on the results and then you conclude in the last sentence that a larger inter-implant distance could have an influence. | We agree with the reviewer that those sentences were slightly misleading and corrected this sentence in the conclusion (line 518-519). |
| **Reviewer 3** | **Response** |
| 1. The authors’ main claim is that children showed significant improvements in speech recognition in quiet and in noise despite a great inter-implant interval. However, this is not explored in the manuscript: changes in children’s outcomes over time are not reported (e.g. what percentage showed improvements, no improvement, or got worse?) and whether this level of improvement was impacted by inter-implant interval has not been explored. The statistical effects of inter-implant interval have not been reported for the two subgroups separately (‘bimodal’ children who wore a hearing aid during this inter-implant interval and ‘unilateral’ children who did not). If the level of benefit and predictive factors of the benefit of sequential implantation is to be truly understood, then the effect of inter-implant interval must be explored, and also reported for each group separately rather than collapsing the effect across the two groups and (potentially erroneously) claiming it is irrelevant to speech outcomes. | We thank the reviewer for the feedback. Based on these comments we decided to add outcomes of the univariable regression analysis on the effect of the inter-implant interval on outcomes for the unilateral and bimodal stimulated groups seperately and included these outcomes in Table 4. Secondly, we added this information to the discussion section (line 449). To be mentioned, we did not analyze the effect of the inter-implant delay with regard to the direction of change in outcomes. This would necessitate categorization of outcomes with loss of power in the analysis whereby the study population was too small to support this.  It should be noted that, in each particular case, a full diagnostic evaluation was performed by a multidisciplinary cochlear implantation team (CI-team) to assess eligibility for a second CI, and therefore the impact of the inter-implant interval was already taken into account preoperatively. Therefore, this factor might have had less of an impact on speech recognition as expected. |
| 2. Developmental effects have not been examined or controlled for in analyses. The sample is heterogenous in terms of age and so confounding factors such as linguistic maturation over time must be reported. Likewise, the effect of age at CI1 on bilateral outcomes has not been reported. Again, this is highly variable in the sample, and is a key factor to consider when evaluating the level of benefit of sequential implantation and predictive factors. | We agree with the reviewer that it is important to take into account the effect of linguistic maturation on the outcome performances. Therefore, we used the word list of the NVA (Dutch Audiology Society) which is adapted to children. As children were at least 5 years of age during testing, the lexical effect of this task was thought to be negligible. Secondly we used the Dutch version of the Peabody Picture Vocabulary Test (PPVT-III-NL) to report the receptive vocabulary. Subsequently raw scores were recalculated as a standard score (age -based), with a population mean of 100 and an +/-1SD of 15, corrected for age in the Standard Score. We added this sentence to the method section to clarify this score (line 232). The PPVT-III-NL is validated with intelligence tests and other vocabulary tests^[1]^. Thirdly we used the subset “recalling sentences” of the Dutch version of the Clinical Evaluation of Language Fundamentals (CELF-4-NL)^[2]^, which test is designed to represent the language proficiency in young children of 5 to 18 years old. Outcomes were reported in norm scores, calculated with a reference score, according to age^[3]^. The CELF-4-NL is validated with other vocabulary tests, including the PPVT-III-NL and secondly corrected for learning effect^[4]^. In this way the reported speech and language outcomes out of this study are corrected for age and so developmental effects have been controlled for in analyses. |
| 3. This is a prospective cohort study that, at first look, appears to employ a longitudinal, repeated-measures design to compare pre- and post-CI2 outcomes to address the main objective of determining the benefit of sequential implantation. However, it is unclear for which participants repeated measurements were collected, or whether different groups were in fact assessed at each time point. This confusion is further compound by the author’s contradictory statement in line 264-265: ‘Between group differences were tested using Wilcoxon signed rank test (for related samples)’. Were two separate cohorts assessed at each time point, or were the same children assessed at pre- and post-CI2? The manuscript should be more transparent about what participants were tested and when, and therefore whether between-group or within-subject statistical analyses methods were employed. | The reviewer pointed out that the manuscript should be more transparent about the measurement collection and comparisons made in the analysis. The data was assessed at two time points. For all participants, the primary and secondary outcomes of the study were measured at baseline (within three months before the second cochlear implantation) and at 12 months after the second cochlear implant (post-operatively). To clarify the data collection to the reader we changed the text in the method section, ‘outcome assessment’ paragraph (line 203-206).  To compare outcomes between the unilateral and bilateral CI condition we used within-subject analysis based on the outcome assessment at two time points. We revised the text in the methods data analysis section to explain this to the reader (line 265-269). |
| 4. Further to the above point, there appears to be much missing follow-up (and/or baseline) data. The authors state that missing data has been handled using multiple imputation. This could mean that missing data has been replaced with the mean or a predicted value. However, the authors provide no information about the methods involved and how the missing data was imputed. For repeated measures analyses, only participants with both baseline and follow-up data should be included. | The reviewer points out that we state that missing data has been handled using multiple imputation. It is indeed mentioned in the method section, ‘in case the data was missing at random’ There the data was not missing at random we didn’t used the multiple imputation. We agree with the reviewer that this part in de method section is confusing. Although it was mentioned in the result section that multiple imputation was not possible (see line 280), we skipped this part in the method section. |
| 5. The total number of participants and the number of data sets analysed is inconsistent throughout the manuscript and difficult to navigate. For example, Figure 1 reports that for CVC in silence post-CI2 n=63, but in Table 4 this is n=61 for the first 4 variables. Furthermore, in Table 4 reports that for CVC in silence post-CI2 use of hearing aid (yes/bimodal = 29, no/unilateral = 13) total n = 42. But in Table 2 CVC in silence outcomes use of hearing aid (bimodal = 33, unilateral = 30) total n = 63. | We thank the reviewer for the feedback and checked the number used for each analyses in the database. By this, we corrected Table 2 and 4 and added the numbers used for each outcome and each analyses. Though, some data was missing (not at random) for several children, such as data on education level or use of hearing aid. Therefore, as an addition, we now provided the missings per outcome and per analysis/comparison in the tables (see Table 2 and 4). |
|  |  |
| *Specific comments:* |  |
| 1. Table 2 – Effects of sequential implantation on CVC speech perception (comparison of ‘bilateral CI’ to ‘CI1’) have been helpfully presented separately for bimodal and unilateral subgroups as well as together. Please do the same for the language outcomes presented in this table (even if the n is small for each subgroup, it is still useful for the reader to see this information) | We changed the table accordingly. |
| 2. Table 2 – Why are the 12-month unilateral CI2 outcomes not reported? Baseline Pre-CI2 is reported but post-CI2 are not, only bilateral outcomes are reported. It seems central to the main objective of the manuscript to understand if implantation of the second ear improved outcomes for this ear, especially for those who wore a hearing aid before implantation. | We specified and changed this table accordingly. |
| 3. Table 2 – condition labels used in column headings are unclear and footnotes are confusing. For improved clarity, please consider using more descriptive labels such as ‘baseline CI1/best-aided’, ‘baseline CI2’, ‘postoperative bilateral’, (and also include ‘postoperative CI2’ as mentioned above) and remove footnotes where possible. These labels should be defined in ‘Outcome assessment’ (line 200) or similar. | We changed the table accordingly, and also added these new column headings in the method section, part ‘outcome assessment’ from line 200. |
| 4. Table 2 – CVC score in quiet ‘Baseline Pre-CI2’: Bimodal (n=13) and unilateral (n=17) gives a total n=30, not n=36 as reported here. What is the discrepancy in the reported numbers? | We noticed the discrepancy in the reported numbers. By double checking the data we corrected the numbers in the tables and reported missings seperately (see Table 2). |
| 5. Table 4 – those with support of speech therapy (‘yes’) had lower outcomes than those without (‘no’). Does this mean that children received speech therapy because they were performing poorly and therefore needed it, whereas ‘good’ performers did not need speech therapy? Without context or interpretation, there is a risk that parents may see this result and think that speech therapy is detrimental to outcomes. | We added a textpart in the paragraph ‘Key findings’ of the Discussion. Some children performed poorly and therefore received speech therapy to improve and stimulate their language abilities, some children not needed it and performed better on outcomes, as shown in Table 4. To clarify this part we deleted the words ‘support of’ and instead use the combination: ‘speech therapy’ and added this information to the discussion section (line 424-427). |
| 6. Table 4 – CELF, use of hearing aid, p value is reported as ‘>0.05’. Please report exact p value consistent with all other reporting of p values in this table | We changed the text in the table accordingly. |
| 7. I understand why separate regression analyses were conducted for each outcome measure (e.g. CVC, PPVT, CELF). However, for each of these separate outcomes, there are multiple predictors that are analysed separately. Therefore, all results reported in Table 4 have not been corrected for multiple comparisons and have not been estimated whilst controlling for covariates. This should be clearly stated. | We agree at this point with the reviewer. There were separate outcomes and there were multiple predictors. Whether or not performing a Bonferroni correction is still a topic of debate. In the most favorable situation we would controlled outcomes for covariates or even would have performed multivariable regression analysis. For this, the studied sample was too small. Therefore we solely performed univariable regression in which the contribution of covariates on the described effects could not be determined. To clarify this to the reader we added this issue to the Method section, see line 270 and discussed this in line 483-485. |
| 8. Why were the effects of predictors on CVC scores in noise not analysed, especially given that this outcome was seen to improve post-CI2? | We agree with the reviewer and added the requested scores to the table (see Table 2). |
| 9. Line 383 – 387 No sig effect of inter-implant delay on CVC words across whole group, but was there an interaction? See next comment. | Based on the remarks above we now added analysis for the unilateral and bimodal stimulated recipients to the table and added outcomes to this part of the results section (line 397-398). |
| 10. S1 Fig 2a/b – Please report the associated statistics (i.e., effect size, p value, and/or regression line equation) and n = for each regression line shown. | We added the requested statistics in the figures, reported in de ‘Supporting information’. |
| 11. I am not familiar with the terms ‘Univariable’ and ‘Multivariable’. When talking about assessing the effect of a single predictor variable on a single outcome variable, the standard term is ‘univariate’ or ‘simple linear regression’. Likewise, the standard term for assessing the effect of several predictor variables on one outcome variable is ‘multivariate’ or ‘multiple linear regression’. Please clarify the analysis method used and use standard terminology throughout. | We understand the confunsion in these terms, which is a long standing and persistent misunderstanding of terminology used in biostatistics. Regrettibly the two terms are often misused. Multivariate analyses refers to a statistical analysis with multiple outcomes (for example multiniminal or ordinal regression), while multivariable analysis is referring to a analysis in which the relative contributions of various factors to a single outcome are assessed. In our case we use multivariable and univariable analysis.  The standard terminology for our analysis is thus multi and uni variable, not variate. Regrettibly the terms are often used in the wrong context.  If the reviewer likes to read more about this, we find this an interesting paper about the misuse of the terms <https://www.ncbi.nlm.nih.gov/pmc/articles/PMC3518362/>. |
| 12. Line 387 ‘Multivariable regression analysis was not performed as no significant associations were tested in univariable regression analysis’. Please be aware that if a predictor is not significant in a univariate analysis, it could still be significant in a multivariate relationship once other effects have been incorporated in the model’s estimates. Consider rephrasing or expanding on why a multivariate regression was not performed on those factors with predictive effects (even if they did not reach significance threshold, e.g. moderate effects of age at CI2 and inter-implant delay). | We agree again with the reviewer and it would be seductive to perform a multivariable regression analysis. Nevertheless our sample size was too small to control the univariable analysis for other (multiple) covariates or perform a multivariable analysis. To underline this to the reader we added this to the discussion section (line 483-485). |
| 13. SSQ ‘quality’ domain significantly improved from baseline to post-CI2. This is important and should be reported in the conclusion and abstract. Subsequently, please correct in abstract ‘No significant effect was seen on language outcomes and SSQ’ as the findings reported do not support this statement. | We thank the reviewer for this encouragement and we changed the text in the abstract and conclusion accordingly. |
| 14. Line 430 – when discussing improvements in the SSQ ‘qualities’ domain, the authors state that ‘This outcome repletes the objective measurements of the speech perception scores in quiet and noise as descripted above’. In fact, the qualities domain most closely reflects/represents quality and effort of listening, rather than speech understanding ability. The ‘speech’ domain is the scale that most closely reflects speech understanding ability. | We agree with the reviewer and changed the text accordingly. |

**Supporting information**

**S1. Fig 2a Regression line between speech perception scores (CVC) in quiet in the bilateral situation and the inter-implant interval**.

β unilateral = 0.44 (*n* = 17); *p* = 0.63. β bimodal = 1.49 (*n* = 21); *p* = 0.34. *Note:* CI1 = first cochlear implant; CI2 = second cochlear implant CVC = consonant-vowel-consonant (speech perception).

**S2. Fig 2b Regression line between speech perception scores (CVC) in noise in the bilateral situation and the inter-implant interval.**

β unilateral = 0.44 (*n* = 17); *p* = 0.63. β bimodal = 1.49 (*n* = 21); *p* = 0.34. *Note:* CI1 = first cochlear implant; CI2 = second cochlear implant CVC = consonant-vowel-consonant (speech perception).

1. Williams KT., Wang JJ. Technical References to the Peabody Picture Vocabulary Test-Third Edition (PPVT-III). *Circ Pines AGS*. 1997;
2. Semel E; Wigg EH; Secord WA. Clinical Evaluation of Language Fundamentals, ed 4 (CELF-4). Toronto: Harcourt Assessment Company; 2003.
3. Semel E; Wigg EH; Secord WA. CELF-4-NL: Clinical Evaluation of Language Fundamentals Dutch version -Manual. Amsterdam: Pearson Assessment and Information B.V.; 2010. 145-164 p.
4. Boons T, Brokx JPL, Dhooge I, Frijns JHM, Peeraer L, Vermeulen A, et al. Predictors of Spoken Language Development Following Pediatric Cochlear Implantation. *Ear Hear.* 2012;33:627–39.
