# Supplementary material for: Benefit of sequential bilateral cochlear implantation in children between 5 to 18 years old: A prospective cohort study
Source: PLoS One. 2022 Jul 28;17(7):e0271497. doi: 10.1371/journal.pone.0271497 (PMC9333257; doi:10.1371/journal.pone.0271497)
Supplement: Attachment — Submitted filename: Response to Reviewers.docx [file pone.0271497.s006.docx]

Dear editor,

We would like to thank you again for the opportunity to resubmit the manuscript entitled ‘Benefit of sequential bilateral cochlear implantation in children between 5 to 18 years old: a prospective cohort study’. We added or changed the specific parts, mentioned in the valuable comments of the reviewers and re-submitted our paper again. Our detailed responses to the reviewers’ comments of the previous submission can be found below.

Sincerely,

On behalf of the author-team,

Drs. W.J. Kleijbergen

| **Reviewer 2** | **Response** |
| --- | --- |
| The data is now easier to follow, but I miss one basic blocks in the discussion. I miss where the methodological choice of the selected test procedures for the outcome are critically discussed in the light of other studies. For example, in your study, CVC was used for speech perception. Other research groups with a similar topic use monosyllable comprehension or tests with meaningful words for speech perception. It has to be discussed why you used CVC especially in the light of the fact that no significant correlations with implant age or inter-implant interval were found. Speech perception with CVC does not mean speech understanding of words.  Likewise, a fundamental discussion of the critical time period of speech development based on scientific principles (e.g. Sharma or Kral) is missing.  It would be good if they put a paragraph like that in the discussion. | We would like to thank the reviewer for the valuable comment to explain the methodological choice of the selected test procedures for the outcome of speech perception and secondly for the encouragement to add an additional part to the discussion about the critical time of period of speech perception.  Firstly, we want to underline that we tested speech perception with meaningful words. These words were monosyllabic and had a CVC-structure. In the Netherlands, this is a standardized test that is used for measuring speech recognition. We feel that the reason that the outcomes were not correlated with age of the second cochlear implantation or inter-implant interval was rather related to the time of onset of severe hearing loss rather than the use of our speech recognition task. To clarify this to the reader that these CVC words were meaningful, we added this to methods section (see line 214).  Secondly, we added a part to the discussion (see line 457-461) were we discuss the fundamental issue of the critical time period of the inter-implant delay and limited device use on auditory brainstem and subsequent cortical maturation to explain our results in more detail. |
| **Reviewer 3** | **Response** |
| 1. When referring to frequency of CI2 use on page 14 and in Table 1&4, please change ‘regularly use’ to ‘frequent use’ to keep the subdivision naming consistent with definitions on page 7 line 197. | We would like to thank the reviewer for the feedback. We changed the text accordingly. |
| 2. Please provide Wilcoxon signed ranks Z test statistic in the results tables and/or in the manuscript text. This will enable the reader and any future systematic reviews to calculate effect sizes. | We added the Wilcoxon signed ranks Z test statistic in Table 2 and 3 accordingly. |
